# Development of a Novel Canine Parvovirus Vaccine Capable of Stimulating Protective Immunity in Four-Week-Old Puppies in the Face of High Levels of Maternal Antibodies

**DOI:** 10.3390/vaccines11091499

**Published:** 2023-09-18

**Authors:** Jacqueline Pearce, Norman Spibey, David Sutton, Ian Tarpey

**Affiliations:** 1 MSD Animal Health, 5831 AN Boxmeer, The Netherlands; 2 CAVAC UK Ltd., Cambridge PE26 2PF, UK; norman@cavacuk.com; 3 MSD Animal Health, Milton Keynes MK7 7AJ, UK; david.sutton@msd.com (D.S.); ian.tarpey@msd.com (I.T.)

**Keywords:** canine parvovirus, maternal immunity, vaccination, vaccine efficacy, puppies

## Abstract

Many highly effective vaccines have been developed to protect dogs against disease caused by canine parvovirus, but despite this vaccine interference by maternally derived antibodies continues to cause immunisation failure. To help overcome this limitation we have developed a novel, recombinant canine parvovirus type 2c vaccine strain, based on the structural and non-structural elements of an established type 2 vaccine. This novel CPV-2c vaccine strain has unique efficacy in the field, it is able to induce sterilising immunity in naïve animals 3 days after vaccination and is able to overcome very high levels of maternally derived antibodies from 4 weeks of age—thus closing the immunity gap to canine parvovirus infection in young puppies. The vaccine strain, named 630a, has been combined with an established canine distemper virus Onderstepoort vaccine strain to produce a new bivalent vaccine (Nobivac DP PLUS), intended to immunise very young puppies in the face of high levels of maternally derived antibody. Here, we describe the onset of immunity and maternal antibody interference studies that support the unique efficacy of the strain, and present overdose studies in both dogs and cats that demonstrate the vaccine to be safe.

## 1. Introduction

Canine parvovirus type 2 (CPV-2) is a major viral pathogen of the domestic dog that causes an acute gastroenteritis with high associated morbidity and mortality, particularly in young puppies. The virus first arose following cross species transfer from a closely related carnivore parvovirus and was recognised as a new disease of dogs in the late 1970s. The virus rapidly spread and evolved within the naïve dog population, establishing a sustained chain of transmission of pandemic proportions. By the end of 1980, the original CPV-2 was completely replaced by a new genetic variant termed CPV-2a, which had five distinct nonsynonymous amino acid changes in the viral capsid (VP) gene. While genetically differentiated from CPV-2 by only a few amino acid substitutions, the changes brought marked biological advantages, most notably a regained ability to infect cats and other carnivore species therefore giving it a broader host range. Successive genetic and antigenic variants, CPV-2b and CPV-2c, evolved over the subsequent years through the further substitution of one or two amino acids in the capsid [1,2,3]. Today, the three CPV variants co-circulate with varying geographical prevalence and the virus has been described in a wide range of domestic, captive, and wild canid, felid, procyonid, and mustelid species [4,5]. 

The acute and devastating nature of the disease is such that global vaccine guideline groups have designated vaccines that confer protection against disease caused by canine parvovirus type 2 as ‘core’ vaccines that every dog should receive independent of circumstance or geographical location [6]. Vaccination is the principal method of controlling disease in domestic dogs and in captive populations of wild carnivores, but despite the ready availability of effective vaccines disease outbreaks continue to occur globally [7,8]. This is in part driven by the high transmissibility and stability of the virus in the environment, and in part due to the interference of maternally derived antibodies with the efficacy of the primary vaccination course in young animals [9]. Passive transfer of maternal antibody in dogs occurs primarily through the uptake of colostrum and, although this protects young puppies against disease, it also interferes with the establishment of active immunity to vaccination in the early weeks, in some cases even beyond the traditional final puppy vaccination at 12 weeks of age, leaving a window of susceptibility to infection that may last up until an animal receives its annual/tri-annual booster vaccination [10,11]. It is this immunity gap that is thought to be the primary reason for the continued outbreaks of disease observed in areas with high vaccination uptake rates [12,13]. 

Despite the potential interference of maternally derived antibodies, vaccination studies conducted for licensure purposes often use puppies born from unvaccinated mothers which are free of maternal antibodies, a situation that is not typical of normal clinical usage. Since interference with immunisation from the presence of maternally derived antibodies is a well established phenomenon, any new vaccine that aims to provide meaningful protection should address this issue. This paper describes the development of a live attenuated canine parvovirus vaccine strain that is able to stimulate protective immunity in puppies from four weeks of age in the face of high levels of maternal antibodies. The strain was constructed using recombinant DNA technology and designed to include the amino acid substitutions that are characteristic of the latest CPV variant, CPV-2c. The genetic analysis of field isolates and vaccine strain 154 [14,15] was used to guide the chosen sequence of the final vaccine strain. Given the extensive safety record of the 154 strain (as used in the current Nobivac vaccine range), the aim of constructing a new vaccine strain was to improve the ability of the vaccine strain to overcome maternal antibody whilst maintaining the genetic backbone of vaccine strain 154 and only introducing those changes in the capsid sequence required to improve efficacy and maintain safety. The rationale being to juxtapose the sequences (structural and non-structural) observed in the 154 vaccine strain with the capsid sequences from current CPV variants.

The safety and efficacy of the new strain was tested by conducting studies in 4-week-old puppies since puppies of this age can have high levels of maternal antibodies which are able to interfere with vaccine efficacy and very young puppies are likely to be more sensitive to any residual pathogenicity present in the vaccine virus than older dogs. Moreover, canine and feline vaccines are routinely administered in combinations in order to reduce the number of injections. This improved CPV vaccine strain was therefore formulated with an existing canine distemper virus Onderstepoort vaccine strain from the Nobivac range. In one of the studies described, an intranasal canine infectious respiratory disease (CIRD) vaccine was also administered at the same time in order to reflect the fact that this combination of vaccines may be advantageous in puppies considered at risk of infectious respiratory disease. In addition, given the close relationship between canine and feline parvoviruses and the fact that cats in the same household as recently vaccinated puppies may be exposed to shed vaccine virus in the environment, the safety of the vaccine strain was also demonstrated in young kittens.

## 2. Materials and Methods

### 2.1. Strain Construction

The genome of the CPV-2 vaccine strain 154 [14] was cloned into a bacterial plasmid using standard laboratory techniques to produce the plasmid p154att. Since the replication mechanism of autonomous parvoviruses proceeds via a double-stranded DNA intermediate, a bacterial plasmid can function as such an intermediate if introduced into susceptible canine cells in culture [16] and produce infectious virus. Therefore, genetic changes to the virus could be made by introducing mutations in the cloned plasmid p154att. To this end, plasmid p630att was constructed using a combination of replicative form hybridisation, nucleotide synthesis, restriction enzyme digestion, and ligation to construct an infectious clone which carries the capsid amino acid changes characteristic of a CPV-2c virus in the genetic background of strain 154 (original CPV-2 type). Specifically, the amino acid substitutions in the viral VP2 capsid protein at positions 87 (Met to Leu), 101 (Ile to Thr), 297 (Ser to Ala), 300 (Ala to Gly), 305 (Asp to Tyr) and 426 (Asn/Asp to Glu) within the background of the original CPV type 2 vaccine strain were encoded. Transfection of canine kidney cells (DK) in culture with p630att gave rise to the virus 630a. The genetic identity of both the plasmid and resultant virus was confirmed by sequencing.

### 2.2. Viruses and Cell Culture

CPV 630a was recovered from canine kidney cells transfected with plasmid p630a. Briefly, sub-confluent monolayers of DK cells were trypsinised and subjected to electroporation. Cells were then re-plated and allowed to recover before further propagating the virus. A virulent CPV-2c field isolate (kindly provided by Prof. C. Buonavoglia, Department of Animal Health and Well-being, Faculty of Veterinary Medicine of Bari, Italy) was used as the challenge virus [15].

The isolation and titration of canine parvovirus strains was as previously described [15,17] using canine fibroblast-like tumour cells (A72) or Crandell Rees feline kidney cells (CRFK) and M6B8 medium (MSD Animal Health) supplemented with 5% foetal bovine serum containing penicillin and streptomycin.

### 2.3. Vaccines and Diluents

Virus CPV 630a was used either as a standalone vaccine strain at a titre of 10^7.2^ TCID_50_/dose for the feline study or lyophilised with the Nobivac Onderstepoort canine distemper virus vaccine strain to produce a DP combination vaccine for dog studies, hereafter called Nobivac DP PLUS (MSD Animal Health). The titres of the distemper virus and parvovirus fractions in Nobivac DP PLUS were 10^5.1^ TCID_50_/dose as a minimum and 10^6.6^ TCID_50_/dose (canine distemper virus) or 10^6.8^ TCID_50_ /dose (canine parvovirus) as a maximum. Overdose studies were conducted with at least ten times the maximum dose and efficacy studies with the minimum dose. 

Solvent for Nobivac DP PLUS (MSD Animal Health, phosphate-buffered water) was used to resuspend the lyophilised Nobivac DP PLUS vaccine. 

Nobivac KC (MSD Animal Health) is a bivalent live attenuated *Bordetella bronchiseptica* and canine parainfluenza virus intranasal vaccine that was administered concurrently with Nobivac DP PLUS to reflect standard veterinary practice.

### 2.4. Serological Analysis

Serum samples were assayed to detect specific CPV-2 antibodies using both haemagglutination inhibition (HAI) and serum neutralisation assays against CPV 2a, CPV 2b, and CPV 2c field strains [14,15,18]. The CPV 2a and CPV 2b strains used were of UK origin and the CPV-2c strains used were of USA origin, each isolate derived from a field case. In the HAI test, each serum sample was tested at two different dilutions and the results for each virus averaged to a final titre and expressed against 4 HA units. In most cases, the results against each virus were averaged to a final titre; however, if a serum sample had an antibody titre below the limit of detection, only the HAI result from the lowest dilution was used. Similarly, if a serum sample had an antibody titre whose endpoint was above the limit of detection, only the HAI result from the highest dilution was used. In the serum neutralisation assay, each serum sample was tested against 100–300 TCID_50_ of virus per well.

### 2.5. Virus Isolation from Rectal Swabs

Since CPV is shed in the faeces, virus isolation from rectal swabs is a standard procedure designed to detect shedding of either vaccine or challenge virus. It is also carried out as an exposure check (in addition to antibody) to confirm that SPF puppies in the efficacy studies have not been inadvertently exposed to infection prior to vaccination and/or challenge. Rectal swabs for virus isolation were taken into 1 ml of M6B8 tissue culture medium that included penicillin and streptomycin and were frozen until required for testing. The swab suspension in each sample tube was defrosted, vortexed well and centrifuged with the swab for 10 min at 1000× *g* at +4 °C to pellet gross debris. Each swab suspension was semi-quantitatively analysed in duplicate by 5-fold dilution in A72 or CRFK cells at the time of plating. The plates were incubated at 37 °C/5% CO_2_ for 3–4 days and analysed by CPV-specific immunofluorescence (IFA). The plates were fixed in methanol followed by staining with an anti-CPV monoclonal antibody [19] and a rabbit anti-mouse FITC conjugate (SIGMA).

### 2.6. Animals

Canine studies were performed in either (i) weaned 4-week-old beagle puppies born to conventionally vaccinated mothers with an up-to-date vaccination history (Nobivac range including CPV-2 vaccine strain 154) obtained from a registered supplier; or (ii) unweaned 4-week-old puppies born to unvaccinated and unexposed mothers from a non-commercial specific pathogen free (SPF) colony. Feline studies were carried out in weaned 11–12-week-old kittens born to unvaccinated and unexposed mothers from a non-commercial specific pathogen free (SPF) colony. Using older kittens (as compared to the 4-week-old puppies) was preferable from a welfare perspective and also reasonable in terms of likely exposure risk. There is no evidence that kittens of 4 weeks of age are more likely to be exposed to vaccine virus, either shed from recently vaccinated puppies in the same household, or following the accidental administration of the vaccine in a veterinary practice.

### 2.7. Ethics Statement

These studies were performed in compliance with Directive 2010/63/EU on the protection of animals used for scientific purposes as transposed into the national law of the applicable country. Prior to the start of each study, each animal was examined and declared to be healthy and suitable for inclusion. The animals were continuously monitored under veterinary care; unvaccinated animals that succumbed to disease were euthanised upon signs of acute malaise in order to reduce animal suffering.

### 2.8. Safety Studies

#### 2.8.1. Study 1: Safety of an Overdose and Repeat Dose of Nobivac DP PLUS in Naïve Four-Week-Old Puppies

A litter of five, unweaned, four-week-old puppies born to an unvaccinated SPF mother were inoculated with a 10× overdose of Nobivac *DP PLUS* followed by a single maximum titre repeat dose 21 days later. The overdose was achieved by reconstitution of 10 maximum titre freeze dried vials of vaccine in Solvent for Nobivac DP PLUS. A commercial dose of Nobivac KC was administered intranasally to each puppy concomitant with the first vaccination. The observation period was concluded 21 days after the second vaccination. 

The puppies were closely examined daily throughout the study for a comprehensive range of clinical parameters to monitor for adverse systemic and local reactions. Any clinical abnormalities were documented and a clinical score assigned, site reactions observed were measured using a caliper. Rectal temperatures were measured before and after each vaccination and daily body weights were taken as a quantitative indicator of clinical health. EDTA blood samples were taken pre- and post- the overdose vaccination to monitor for leukopenia.

#### 2.8.2. Study 2: Safety of a High Titre Dose of CPV 630a in Naïve 11–12-Week-Old Kittens

Fifteen domestic short-haired SPF cats of 11–12 weeks of age were divided into two groups. Group 1 comprised ten animals and Group 2 comprised five animals; all animals were housed together in one room. Group 1 received two high titre doses of 10^7.2^ TCID_50_ CPV 630a via the subcutaneous and oronasal routes. The rationale for dosing via both these routes was to reflect the possible routes of exposure—oronasal for contact with shed virus from recently vaccinated puppies and subcutaneous to reflect the possibility of accidental injection of a dose of vaccine. Group 2 remained unvaccinated sentinels and were included to detect whether naïve in-contact kittens could become naturally infected with vaccine virus shed from the Group 1 kittens. The cats were examined daily for 21 days post-vaccination for a comprehensive range of clinical parameters to monitor for adverse reactions. Any clinical abnormalities were documented and a clinical score was assigned. Daily rectal temperatures and body weights were taken as a quantitative indicator of clinical health. Blood samples were taken pre- and post-vaccination to monitor for leukopenia and to assess antibody responses. Rectal swabs were taken and assayed to determine the presence of virus on CRFK cells.

### 2.9. Efficacy Studies

#### 2.9.1. Study 3: Onset of Immunity in Naïve Four-Week-Old Puppies

Twelve four-week-old SPF puppies from two separate litters were assigned to two groups: a group of seven puppies (Group 1) and a group of five puppies (Group 2). The puppies remained in their litter groups with their mothers and were housed in separate rooms under biocontainment. All the dogs were declared fit and healthy by veterinary inspection and shown to be negative for CPV by swab and serology at the start of the experiment. Each puppy in Group 1 was vaccinated with a single minimum titre dose of Nobivac DP PLUS reconstituted in solvent for Nobivac DP PLUS (10^5.1^ TCID_50_ /dose for each component). The puppies in Group 2 remained unvaccinated. Three days post vaccination, each puppy was orally challenged with 10^5.4^ TCID_50_ virulent CPV-2c strain of Italian origin. To facilitate experimental canine parvovirus infection a short period of fasting consonant with the age of the animals was applied, the mothers and any solid food in the room were removed 4 h prior to challenge and returned 2 h after challenge. Water was available throughout.

Blood samples were taken on the day of challenge (three days after vaccination in the vaccinated group) to measure the developing serological response to vaccination, and additionally, 7 and 14 days after challenge to measure the serological response to vaccination and/or infection. EDTA blood samples for circulating white blood cell counts were taken pre-and post-challenge to monitor for leukopenia. Rectal swabs for virus isolation were taken daily to evaluate the shedding of vaccine or challenge virus on A72 cells. All dogs were closely monitored in the post-challenge period, including the daily assessment of clinical signs, and the measurement of rectal temperatures and body weight.

#### 2.9.2. Study 4: Efficacy in Four-Week-Old Puppies in the Presence of Maternally Derived Antibody

Sixteen four-week-old puppies from three litters were assigned to two groups: a group of 11 puppies (Group 1) and a group of five puppies (Group 2). All three mothers were conventionally vaccinated (initially as puppies and then annually boosted using a commercial CPV-2 type vaccine) and their puppies had moderate to high levels of maternal derived antibody against canine parvovirus that were representative of a field situation. The puppies were pre-screened to measure their maternal antibody levels and allocated to one or othergroup in order to ensure a similar spread of MDA across both groups. Since some time was needed to carry out the testing, the MDA titre measurements from a few days before the day of vaccination were used to predict the actual MDA titres on the day of vaccination. Each group was housed in a separate biocontained room. Rectal swabs were taken to confirm the absence of virus shedding prior to vaccination. Each puppy in Group 1 was vaccinated with a single minimum titre dose of Nobivac DP PLUS (10^5.1^ TCID_50_/dose for each component). The puppies in Group 2 were not vaccinated and acted as controls for the decline of maternally derived antibody. On the day of vaccination, but immediately prior to vaccination, blood samples were taken from each puppy to determine the level of MDA. Blood samples were taken on the day of vaccination and then every three or four days until 59 days post vaccination to determine the effect of vaccination in Group 1 and to track the decline of maternally derived antibody in Group 2.

## 3. Results

### 3.1. Study 1: Safety of an Overdose and Repeat Dose of Nobivac DP PLUS in Naïve Four-Week-Old Puppies

#### 3.1.1. Clinical Observations

All of the puppies remained in excellent health throughout the observation period and did not present with any abnormal clinical signs that could indicate a systemic reaction to vaccination. There was no increase in body temperature following either the 10× overdose or maximum titre repeat dose vaccinations and all of the dogs maintained a largely unbroken increase in weight throughout the study, putting on weight as would be predicted for puppies of that age (unpublished data). There were occasional days on which a puppy lost weight compared with the previous day, but the loss in weight was minimal and did not exceed 30 g. As there was no indication of a reduction in appetite such a transient weight loss in small puppies is likely to be due to the difference between a full or empty stomach/bowel from one day to the next.

In the days immediately following each vaccination some of the puppies were observed with a transient local reaction at the injection site. One puppy presented with a soft non-measurable diffuse thickening of the skin around the injection site on days 2 and 3 post 10x overdose. Three different puppies presented with a similar soft non-measurable diffuse thickening on the day after the maximum titre repeat dose that lasted for one or two days.

#### 3.1.2. Serology and white blood cell counts

In keeping with their SPF status and derivation from unvaccinated mothers none of the puppies had any detectable antibodies to canine parvovirus prior to vaccination; in addition, puppies were also shown to be negative with regard to antibodies against canine distemper virus (unpublished data). The data in Table 1 show that a 10× overdose vaccination of Nobivac DP PLUS had little if any effect on the circulating white blood cell counts and no evidence of a significant diminution or leukopenia was observed.

### 3.2. Study 2: Safety of a High Titre Dose of CPV 630a in Naïve 11–12-Week-Old Kittens

#### 3.2.1. Clinical Observations

All cats were observed daily for a range of clinical signs, but no cat recorded any clinical score throughout the study. There was no indication of pyrexia, and other than some minor fluctuations in weight between consecutive days, all cats gained body weight as would be expected between the onset and close of the study. 

#### 3.2.2. White Blood Cell Counts and Viral Shedding

Previous studies have suggested that viral shedding may coincide with a drop in white blood cell counts in CPV infected cats [20]. In this study, the white blood cell levels in the majority of vaccinated cats dipped on day 4 after vaccination but hadbegun to return to basal levels by day 6. This coincided with a period of shedding between days 3 and 5 (Table 2). There was one vaccinated cat which tested positive on both days 4 and 11. The positive isolation on day 4 coincided with a drop in white blood cell count so likely represents active replication of vaccine virus; however, the later positive on day 11 is not associated with any similar drop. A likely explanation for the day 11 isolation is therefore that this cat had ingested shed virus in the environment which had passed through the intestine by simple mechanical transit and was by this means present in the faeces. Virus was isolated from one of the Group 2 sentinel cats showing that a sufficient virus was being shed for inter-animal transmission.

The baseline mean value was calculated for each cat using counts from days −4, −2, −1, and 0. 

### 3.3. Study 3: Onset of Immunity in Naïve Four-Week-Old Puppies

#### 3.3.1. Clinical Observations

No clinical observations or deviations in core body temperature were recorded in any of the vaccinated puppies following challenge and they remained in good health. The vaccinated puppies showed a steady increase in body weight throughout the study, although one puppy did show a temporary dip in recorded body weight 6 days post challenge (unpublished data). This transient loss was rapidly regained by the next day. In notable contrast, all control puppies began to exhibit pathognomonic signs of canine parvovirosis together with a marked check in weight gain from 4 days after challenge from which they did not recover. The signs of parvovirosis included malaise, reduced appetite, dehydration, vomiting, a poor general condition, and profuse mucoid diarrhoea that became bloody. Rectal temperatures increased to ≥39.5 °C during the early stages of disease progression before dropping to between 37 °C and 38 °C as the humane endpoint was reached.

#### 3.3.2. Serological Responses

The serological response of the puppies to vaccination and challenge by both haemagglutination inhibition assay (HAI) and serum neutralisation (SN) is shown in Table 3. In keeping with their SPF status and derivation from unvaccinated mothers all of the puppies were seronegative for canine parvovirus prior to the start of the study. Following vaccination, the puppies in Group 1 began to show evidence of active seroconversion on day 3 (the day of challenge) and had developed antibody levels approaching maximum levels on day 6. The control group did not show any serological response to challenge until the terminal bleed was taken on day 8 or 9 (5–6 days post challenge), at which time disease progression was quite marked. 

After vaccination, although both the SN and HAI antibody titres increased, the SN titres were somewhat slower to rise and consequently lower on the initial two post-vaccination timepoints (days 3 and 6) than they were after active seroconversion was established by day 10. This suggests that the nascent antibody pool had a relatively low neutralisation capacity and that the humoral response was still in the process of undergoing expansion and affinity maturation until sometime between days 6 and 10 post-vaccination. 

#### 3.3.3. Viral Shedding

Virus was isolated from the rectal swabs to determine the presence or absence of infectious vaccine or challenge virus (Table 4). All of the vaccinates shed vaccine virus for a period of between one and five days from 2 to 6 days post vaccination, as would be expected following vaccination with a live attenuated canine parvovirus vaccine strain. The controls remained negative until day 6 or 7, three to four days post-challenge, after which each puppy shed high levels of infectious field virus until the humane endpoint was reached. Since one vaccinated puppy was still shedding virus on day 6, a day that overlapped with the onset of field virus shedding in the controls, the swabs from each animal in the study were evaluated in a diagnostic PCR using primer sets specific for vaccine virus, field virus, or universal canine parvovirus. All the vaccinated puppies were shown to be positive for vaccine virus shedding and were shown to be free of challenge virus. The control animals in contrast were all positive for challenge virus and were negative for vaccine virus. All samples were positive for universal canine parvovirus (unpublished data). This confirms that shedding of challenge virus could not be detected from any of the vaccinates.

#### 3.3.4. White Blood Cell Counts

Following exposure to field strains of canine parvovirus, virus replication begins in the secondary lymphoid tissue of the oropharynx and mesenteric lymph nodes and the virus disseminates to the thymus where it replicates in the rapidly dividing lymphocytes [20]. Viral replication and apoptosis cause thymic depletion and immunosuppression through lymphocytolysis which can be quantified by means of the circulating white blood cell counts post-infection. Leukopenia is considered to be a diminution of the white blood cell levels below 50% of the baseline mean.

Following challenge of the vaccinated puppies there was a transient dip in the white blood cell levels three days post-infection which, in the majority of cases, had rebounded to basal levels by five days post-infection (Table 5). These results demonstrate that vaccination is able to prevent leukopenia following field virus exposure. In contrast, the white blood cell measurements in the control animals steadily declined as the infection took hold and leukopenia was evident in one puppy as it reached the humane endpoint. 

### 3.4. Study 4: Efficacy in Four-Week-Old Puppies in the Presence of Maternally Derived Antibody

The three conventionally vaccinated bitches from which the puppies were selected had high levels of circulating antibodies to canine parvovirus and their puppies had a moderate to high range of maternally derived antibody levels that were evenly divided across the two groups (Table 6 and Supplementary Table S1). On the day of vaccination, the maternal antibodies against CPV-2c in the Group 1 puppies ranged from 288 to 1664 HAI units and the maternal antibodies against CPV-2c in the Group 2 control puppies ranged from 288 to 832 HAI units (the day 0 titres in Table 6). 

Following vaccination, all Group 1 pups actively seroconverted within a period of 8–51 days, and prior to the decay of maternally derived antibody in the controls. Depending on the timing of the bleed, the moment of maternal antibody sequestration by viraemic CPV 630a virus can be seen in abruptly seronegative samples that follow a timepoint positive for maternal antibody and precede a timepoint with evident active seroconversion; for example, puppy 8584 on Day 7 (Table 6). Following active seroconversion, all antibody titres remained high throughout the remainder of the study. The maternal antibody levels within the control puppies declined in a similar manner over time, with a half-life of approximately 11–14 days. 

## 4. Discussion

Parvoviruses are small non-enveloped DNA viruses that are extremely resistant to environmental conditions [21]. Due to their robust physical nature they are notoriously difficult to eliminate from kennels and breeding establishments and consequently young puppies can be exposed to virus from birth onwards. Maternally derived antibodies (MDA) above a certain threshold are able to protect puppies from disease; however, these antibodies can also interfere with vaccination. Levels of MDA often differ markedly both between puppies of the same litter and between puppies of different litters, the reasons for which are multifactorial and may depend on a combination of the breed involved, the serological status of the bitch, the method of parturition, and the management of periparturient bitch and litter [22]. The mean half-life of CPV MDA has been reported to be 9–10 days [23]; however, this is not an extensively researched area and more recent studies have shown a mean half-life of 13.5 days and 13.4 days for CPV MDA [24,25]. Irrespective of the overall mean value, it is important to stress that, in an individual animal, predicting the precise level of acquired immunity and the precise rate of decline is not possible. As puppies are often weaned at 7–8 weeks of age and then separated from their mothers this is the age at which first vaccinations are usually given. However, this is not a universal practice and puppies may be separated at a younger or older age. Furthermore, since neither the antibody level of the bitch nor the levels of MDA in a puppy are known in a normal clinical setting, the level of individual risk posed by parvoviral MDA interference is also unknown. The period of time between a puppy becoming susceptible to infection, due to the decline of MDA, and the time at which the first vaccination is effective has been termed the ‘immunity gap’ or ‘window of susceptibility’ [26]. Therefore, the development of a vaccine and vaccination schedule that will reduce this gap to the shortest time possible or eliminate it is a major aim in canine vaccine development. Here, we present a novel CPV-2c vaccine virus that is able to overcome very high levels of maternally derived antibody in puppies as young as 4 weeks of age, induce sterilising immunity in naïve animals three days post vaccination, and is demonstrably safe in both dogs and cats. To date, this degree of parvoviral efficacy is unprecedented and not equalled by other canine parvovirus vaccine strains available on the market (paper in review).

In order to demonstrate the efficacy of the strain in the presence of MDA, four-week-old puppies were selected to have high levels of maternal antibodies compared with others; a range of between 288 to 1664 HAI units (806 to 4524 SN_50_) as measured against type 2c. Efficacy in this study was measured by breakthrough antibody response rather than challenge since MDA itself will affect the outcome in a challenge study. Even though these puppies were not challenged, we can nonetheless conclude that the observed serological responses were indicative of protective immunity. It is well established that antibody titres against CPV are tightly correlated with clinical protection. The immune response is primarily driven by the humoral arm of the immune system and titres of ≥80 HAI units are considered protective [23]. Following vaccination, all the vaccinated puppies developed a robust immune response to vaccination (Table 6 and Supplementary Table S1). Each of the vaccinated puppies generated an active immune response at a different time point and it is interesting to note that the serological response to CPV in each case was preceded by a temporary dip in the antibody titre to an unmeasurable level. This transient drop has been observed by the authors in other in vivo MDA studies and correlates with a viraemia (internal data), presumably indicating the sequestration of maternal antibodies by the replicating vaccine virus. These data demonstrate that, rather than being neutralised, the 630a vaccine strain is able to productively persist and replicate in the presence of maternally derived antibodies. It is tempting to speculate that the virus is residing at an immune privileged site, releasing newly replicated virus into the circulatory system. Future work therefore aims to elucidate the mechanism behind this characteristic. 

Together with the ability to overcome MDA, a rapid onset of immunity is a key attribute in a canine parvovirus vaccine that is intended to provide early and effective protection. The onset of immunity study presented here was performed in antibody free (SPF) vaccinated puppies that were challenged with virulent CPV-2c three days post vaccination, with a non-vaccinated age matched group used as controls. The data from this study provide clear evidence of the rapid immunising capability of strain 630a in maternal antibody negative puppies. None of the vaccinated puppies exhibited any clinical signs of parvoviral disease, they continued to grow and gain weight and there were no signs of pyrexia. No field virus could be detected in the rectal swabs taken from the vaccinated group, indicating the rapid induction of a sterilising immune response. Given the environmental stability of canine parvovirus in an establishment, a rapid onset of immunity is of great value in controlling an outbreak situation. 

The safety of any new vaccine strain in the target animal is of paramount importance, but this is particularly true of one that will be administered to young animals and that may be shed in the environment to potentially infect other productive hosts. Given the susceptibility of cats to CPV-2c strains and the likelihood of their living within the same household as dogs, the safety of the 630a vaccine strain in cats was an important requirement. Here, we present data to show that a 10-fold overdose of the vaccine strain is safe when administered to naïve four-week-old puppies concurrently with an intranasal kennel cough vaccine, or when administered to naïve cats of 11–12 weeks of age. In common with most effective live canine parvovirus vaccine strains [27], the 630a strain was recovered from rectal swabs indicating that the virus had undergone replication in the gut. However, despite their young age and complete lack of maternal antibodies, none of the animals exhibited any clinical signs such as diarrhoea, vomiting, malaise, or pyrexia. Leukopenia is often a consequence of parvoviral infection in both cats and dogs [20], and is therefore a criterion by which both safety and efficacy can be assessed. The safety studies described here in cats and in very young puppies demonstrate that CPV 630a is safe in this respect, although three of the vaccinated cats showed a transient dip in white cell counts correlated with a limited degree of virus shedding. These data show that the CPV vaccine strain does not possess any residual pathogenicity as a result of the introduction of the type 2c amino acid changes in the viral capsid. The strain has also been shown to maintain genetic stability and not to revert to virulence after five sequential passages through groups of naïve dogs (unpublished data).

In summary, a novel CPV vaccine strain has been developed and combined with an established CDV component. The resulting bivalent vaccine is intended to be used in very young puppies, typically between 4 and 6 weeks of age. Despite the proven ability of the CPV component in stimulating active immunity in the face of high levels of MDA at this age, it is not intended to remove the need for later vaccination doses. Following the use of this vaccine, puppies should continue to receive their normal post-weaning primary course of core and non-core components in accordance with manufacturer’s advice and international vaccination guidelines.

## 5. Conclusions

The data presented here demonstrate that canine parvovirus vaccine strain 630a is both safe and highly efficacious in puppies from 4 weeks of age onwards and will provide a valuable addition to the canine vaccine portfolio. It is able to overcome high levels of maternally derived antibodies and therefore provide protection to puppies at their most vulnerable age.

## Figures and Tables

**Table 1:** Total white blood cell counts.

Pup ID	Days Prior to Vaccination			Baseline Mean	Days Post Vaccination			
	−4	−2	0		+3	+5	+7	+10
6825	8.4	12.4	13.0	11.3	12.1	8.6	10.3	22.1
6100	8.4	10.7	11.6	10.2	8.8	8.2	9.8	21.6
7800	13.3	12.7	16.6	14.2	11.5	9.3	12.9	25.5
6711	6.9	6.8	7.8	7.2	6.9	5.9	10.4	15.2
9652	8.9	12.6	11.4	11.0	10.0	9.8	10.9	24.8

Counts are given in 10^9^ cells/L.

**Table 2:** White blood cell counts and virus shedding.

Cat ID	Group	Baseline Mean	Days Post-Vaccination				Days on Which Virus Shed
			Day 4	Day 6	Day 8	Day 10	
35992	1 Vaccinates	23.9	15.5	17.8	25.0	29.1	4, 5
36060		22.8	11.1	13.8	17.6	18.5	4, 11
36135		20.6	16.5	14.7	17.3	20.4	5
36137		15.7	13.6	15.1	17.6	18.0	-
36372		22.0	*8.7*	15.3	24.1	30.3	3
36380		34.1	23.3	23.4	28.2	24.5	-
36414		16.1	9.3	10.7	15.0	15.7	1
36415		27.9	13.2	22.7	21.9	22.0	5
36416		16.1	19.5	24.2	24.4	31.4	-
36467		19.3	10.8	13.1	20.2	23.5	
35971	2 Sentinels	17.3	18.7	18.4	22.1	20.4	11
36210		21.0	18.2	25.3	23.3	20.9	-
36356		15.9	18.0	18.3	21.2	15.2	-
36490		25.1	22.3	24.9	27.4	20.1	-
36365		23.5	26.0	25.9	25.5	29.1	-

Counts are given in 10^9^ cells/L.

**Table 3:** Serological response to vaccination and challenge.

Group	Dog ID	Days Post Vaccination and Challenge						
		0 Vaccination	3 Challenge		6		10	
		HAI	HAI	SN	HAI	SN	HAI	SN
1 Vaccinates	5963	16 ^c^	288	≤14	40,960	8300	>40,960	43,632
	6286	16 ^c^	144	≤16	40,960	4525	>40,960	37,807
	6914	8 ^c^	224	≤14	26,624	10,973	18,432	21,816
	1077	<8	288	≤16	40,960	15,020	>40,960	41,587
	1611	<8	144	≤14	40,960	15,020	>40,960	32,254
	5583	8 ^c^	288	≤14	40,960	6400	>40,960	30,444
	6946	<8	288	≤14	>36,864	10,908	>40,960	25,600
2 Controls	6937	<8			<8		9216 ^a^	2691 ^a^
	2134	<8			<8		9216 ^a^	2727 ^a^
	6412	<8			<8		4608 ^a^	2016 ^a^
	7286	16 ^c^			<8		9216 ^a^	5198 ^a^
	6013	16 ^c^			<8		4608 ^b^	1270 ^b^

^a^ Samples taken early on day 9 (6 days after challenge) as the humane endpoint was applied; ^b^ Samples taken late on day 8 (5 days after challenge) as the humane endpoint was applied; ^c^ Serum interference in the first or second well of the HAI plate, this is not unusual when using low pre-dilutions of sera from young pups.

**Table 4:** Canine parvovirus rectal swab isolations: viral load estimations (log_10_ TCID_50_/swab).

Group	Dog ID	Days Post-Vaccination and Challenge											
		−1	0 (V)	1	2	3 (C)	4	5	6	7	8	9	10–17
1 Vaccinates	5963	-	-	-	≤2.1	≤2.1	≤2.45	2.10	-	-	-	-	-
	5583	-	-	-	1.75	2.45	≤2.1	≤2.1	-	-	-	-	-
	6286	-	-	-	-	-	-	≤2.45	-	-	-	-	-
	6946	-	-	-	-	2.80	2.45	2.80	-	-	-	-	-
	6914	-	-	-	-	2.45	3.49	2.80	-	-	-	-	-
	1077	-	-	-	-	2.10	2.80	2.45	-	-	-	-	-
	1611	-	-	-	≤2.1	≤2.45	3.15	3.84	2.52	-	-	-	-
2 Controls	6937	-	-	-	-	-	-	-	3.15	6.29	≥6.64	≥6.64	†
	2134	-	-	-	-	-	-	-	≤2.1	≥6.64	≥6.64	≥6.64	
	6412	-	-	-	-	-	-	-	3.84	≥6.64	≥6.64	≥6.64	
	7286	-	-	-	-	-	-	-	-	≥6.64	≥6.64	≥6.64	
	6013	-	-	-	-	-	-	-	2.45	≥6.64	≥6.64	†	

V: Day of vaccination, C: Day of challenge, -: No virus could be isolated, †: Euthanised at the humane endpoint.

**Table 5:** Circulating white blood cell counts.

Group	Pup ID	Days Pre- and Post-Challenge							
		−4	−2	0 (C)	Baseline Mean	+3	+5	+7	+10
**1** **Vaccinates**	**5963**	14.9	15.6	7.7	**12.7**	6.9	10.2	15.0	11.6
	**6286**	12.2	13.1	9.9	**11.7**	6.7	7.5	15.1	10.4
	**6914**	8.5	10.4	9.5	**9.5**	7.5	9.7	14.8	11.8
	**1077**	7.6	9.4	6.7	**7.9**	5.1	8.3	12.3	11.0
	**1611**	11.4	13.3	10.1	**11.6**	7.9	6.0	14.2	9.6
	**5583**	3.5	9.1	7.9	**6.8**	*	8.7	13.7	9.9
	**6946**	8.6	10.5	7.2	**8.8**	6.5	7.9	15.1	11.3
**2** **Controls**	**6937**	8.2	7.6	7.6	**7.8**	8.8	5.7	†	
	**2134**	7.3	7.3	7.5	**7.4**	7.3	5.5		
	**6412**	10.8	12.1	11.8	**11.6**	10.0	7.4		
	**7286**	7.3	8.4	6.6	**7.4**	7.1	***3.6***		
	**6013**	8.2	8.5	8.3	**8.3**	6.4	6.0		

Counts are given in 10^9^ cells/L. * EDTA blood sample clotted, measurement not possible, C: Day of challenge (study day 3). The baseline mean value was calculated for each pup using counts from days −4, −2, and 0 (**bold**). Total white blood cell counts that dropped below 50% of the basal average are shown in ***bold italics***. † Euthanised on welfare grounds

**Table 6:** Serological analysis.

Group	Bitch ID	Bitch Titre HAI 2c	Pup ID	Age on Day 0	HAI 2c Titre of the Pups on Day:															
					0	4	7	11	14	18	21	25	28	31	34	38	41	45	48	52
**1** **Vaccinates**	**6D51183D30**	4608	8995	4wk 3d	288	288	288	288	104	9216	18,432	18,432	13,312	13,312	18,432	18,432	13,312	18,432	13,312	14,336
	**6D4C6A4D5D**	4608	9019	4wk 4d	288	288	288	288	<16	18,432	18,432	9216	6656	9216	9216	9216	6656	13,312	9216	9216
	**6D4C6A4D5D**	4608	8651	4wk 4d	416	288	208	9216	9216	13,312	18,432	9216	6656	9216	18,432	14,336	13,312	>26,624	18,432	18,432
	**6D51183D30**	4608	9030	4wk 3d	416	208	288	416	9216	18,432	18,432	18,432	13,312	13,312	18,432	14,336	13,312	18,432	13,312	9216
	**6D4C6A4D5D**	4608	8580	4wk 4d	416	416	<16	6656	†											
	**6D4C6A4D5D**	4608	8584	4wk 4d	416	288	<16	6656	6656	9,216	9216	9216	6656	6656	13,312	9216	9216	18,432	18,432	9216
	**6D4D03500D**	9216	8635	4wk 1d	832	576	896	576	576	416	288	208	144	104	144	72	<16	>40,960	28,672	18,432
	**6D4D03500D**	9216	8732	4wk 1d	832	576	576	416	416	288	288	144	1152	40,960	40,960	26,624	18,432	>26,624	13,312	18,432
	**6D4D03500D**	9216	8998	4wk 1d	576	576	576	832	9216	13,312	9216	9216	6656	6656	9216	9216	6656	18,432	9216	9216
	**6D4D03500D**	9216	9002	4wk 1d	832	576	576	576	576	416	288	208	144	104	144	72	52	144	52	18,432
	**6D4D03500D**	9216	8755	4wk 1d	1664	576	1152	832	416	416	288	208	144	144	144	72	72	104	36	13,312
**2** **Controls**	**6D51183D30**	4608	9033	4wk 3d	288	144	288	208	104	208	72	52	72	36	56	52	18	52	52	<8
	**6D4C6A4D5D**	4608	9024	4wk 4d	416	288	288	288	208	208	144	104	72	72	112	52	36	72	36	18
	**6D4D03500D**	9216	8747	4wk 1d	832	576	832	576	416	416	288	144	144	104	144	52	52	104	36	26
	**6D4D03500D**	9216	8582	4wk 1d	832	576	832	576	416	416	288	144	144	144	144	72	52	104	36	36
	**6D4D03500D**	9216	8751	4wk 1d	832	416	832	416	288	416	288	144	112	104	144	36	36	72	36	18
Antigen HA units					4HA	4HA	4HA	4HA	4HA	4HA	4HA	4HA	4HA	4HA	4HA	4HA	4HA	4HA	4HA	4HA

†: Pup was euthanised for general health reasons unrelated to vaccination, Grey shaded: Active seroconversion (>two-fold rise in antibody titre). The first indication of an active immune response to vaccination is marked by the sudden disappearance of maternally derived antibody and a short timeframe of seronegativity that coincides with a vaccine virus viraemia and precedes a rapid active antibody response.

## Data Availability

Not applicable.

## Supplementary Materials

The following are available online at https://www.mdpi.com/article/10.3390/vaccines11091499/s1, Table S1: (A): MDA efficacy hemagglutination inhibition (HAI) serological data; (B): MDA efficacy serum neutralisation (SN) serological data.
