# Supplementary material for: Development of a Novel Canine Parvovirus Vaccine Capable of Stimulating Protective Immunity in Four-Week-Old Puppies in the Face of High Levels of Maternal Antibodies
Source: Vaccines (Basel). 2023 Sep 18;11(9):1499. doi: 10.3390/vaccines11091499 (PMC10534519; doi:10.3390/vaccines11091499)
Supplement: Supplementary file 1 [file vaccines-11-01499-s001.zip › vaccines-2577175-supplementary.pdf]

TABLE S1A. MDA Efficacy Study Haemagglutination Inhibition (HAI) Serological Data

Group	Pup ID	Days Post Vaccination																	
		0			4			7			11			14			18		
		2a	2b	2c	2a	2b	2c	2a	2b	2c	2a	2b	2c	2a	2b	2c	2a	2b	2c
1 Vaccinates	**8732**	576	576	832	576	576	576	832	576	576	416	288	416	288	288	416	288	288	288
	**8995**	288	288	288	288	288	288	256	288	288	208	208	288	144	144	104	7168	9216	9216
	**9019**	288	288	288	208	288	288	288	288	288	288	208	288	<16	<16	<16	14,336	18,432	18,432
	**8651**	288	288	416	288	416	288	144	144	208	4608	6656	9216	4608	9216	9216	9216	13,312	13,312
	**8580**	416	416	416	288	416	416	16	<16	<16	3328	4608	6656	†					
	**9030**	288	288	416	288	288	208	208	288	288	288	416	416	4608	6656	9216	18,432	13,312	18,432
	**8584**	416	416	416	416	416	288	18	<16	<16	3328	3328	6656	4608	6656	6656	9216	9216	9216
	**9002**	576	832	832	576	576	576	576	576	576	576	288	576	416	416	576	576	448	416
	**8998**	832	832	576	576	576	576	576	576	576	576	576	832	4608	6656	9216	9216	9216	13,312
	**8635**	1152	1152	832	576	832	576	576	576	896	576	576	576	576	672	576	416	576	416
	**8755**	1152	1152	1664	1152	832	576	1152	832	1152	576	576	832	576	576	416	576	416	416
2 Controls	**9033**	208	288	288	144	208	144	144	208	288	208	144	208	144	208	104	144	144	208
	**9024**	416	288	416	288	288	288	288	416	288	288	288	288	208	288	208	288	208	208
	**8747**	832	832	832	576	576	576	576	576	832	576	416	576	416	288	416	288	288	416
	**8582**	832	832	832	576	576	576	576	832	832	832	576	576	576	576	416	576	448	416
	**8751**	832	576	832	576	576	416	832	576	832	576	416	416	288	416	288	416	288	416

Group	Pup ID	Days Post Vaccination														
		21			25			28			31			34		
		2a	2b	2c	2a	2b	2c	2a	2b	2c	2a	2b	2c	2a	2b	2c
1 Vaccinates	8732	144	144	288	104	144	144	576	832	1152	18,432	>40,960	>40,960	>40,960	>40,960	>40,960
	8995	18,432	18,432	18,432	18,432	18,432	18,432	13,312	18,432	13,312	9216	13,312	13,312	18,432	18,432	18,432
	9019	9216	13,312	18,432	9216	9216	9216	6656	9216	6656	6656	9216	9216	6656	9216	9216
	8651	9216	18,432	18,432	6656	9216	9216	6656	9216	6656	6656	13,312	9216	9216	18,432	18,432
	8580								†							
	9030	13,312	18,432	18,432	18,432	18,432	18,432	13,312	18,432	13,312	6656	13,312	13,312	13,312	18,432	18,432
	8584	6656	9216	9216	4608	9216	9216	6656	9216	6656	6656	9216	6656	9216	9216	13,312
	9002	288	288	288	144	208	208	144	144	144	104	104	104	104	104	144
	8998	6656	9216	9216	4608	6656	9216	4608	9216	6656	4608	6656	6656	9216	9216	9216
	8635	288	288	288	208	224	208	144	144	144	144	104	104	104	72	144
	8755	288	288	288	208	144	208	144	144	144	144	104	144	104	144	144
2 Controls	9033	52	72	72	36	52	52	72	72	72	26	26	36	18	36	56
	9024	144	104	144	72	144	104	104	104	72	72	52	72	72	72	112
	8747	288	144	288	144	144	144	144	144	144	104	104	104	72	72	144
	8582	288	288	288	144	208	144	144	144	144	144	104	144	72	72	144
	8751	208	208	288	144	144	144	144	72	112	72	72	104	72	72	144

Group	Pup ID	Days Post Vaccination														
		38			41			45			48			52		
		2a	2b	2c	2a	2b	2c	2a	2b	2c	2a	2b	2c	2a	2b	2c
1 Vaccinates	8732	18,432	18,432	26,624	13,312	18,432	18,432	13,312	18,432	>26,624	13,312	13,312	13,312	7168	9216	18,432
	8995	9216	9216	18,432	9216	18,432	13,312	13,312	18,432	18,432	13,312	13,312	13,312	9216	9216	14,336
	9019	6656	9216	9216	6656	9216	6656	9216	13,312	13,312	9216	9216	9216	4608	9216	9216
	8651	9216	9216	14,336	13,312	13,312	13,312	18,432	18,432	>26,624	18,432	13,312	18,432	9216	13,312	18,432
	8580								†							
	9030	9216	9216	14,336	9216	9216	13,312	13,312	18,432	18,432	13,312	9216	13,312	9216	9216	9216
	8584	9216	9216	9216	9216	6656	9216	9216	18,432	18,432	9216	9216	18,432	9216	9216	9216
	9002	72	144	72	72	72	52	72	112	144	56	72	52	9216	9216	18,432
	8998	6656	9216	9216	4608	6656	6656	6656	9216	18,432	13,312	7168	9216	4608	6656	9216
	8635	104	104	72	<16	<16	<16	>26,624	>40,960	>40,960	18,432	18,432	28,672	13,312	13,312	18,432
	8755	104	104	72	72	72	72	72	104	104	36	72	36	9216	8192	13,312
2 Controls	9033	36	52	52	28	28	18	16	36	52	36	104	52	<8	<8	<8
	9024	52	52	52	36	36	36	36	72	72	36	104	36	14	18	18
	8747	72	104	52	72	52	52	72	104	104	52	56	36	26	36	26
	8582	72	104	72	72	52	52	72	104	104	72	56	36	36	36	36
	8751	72	52	36	36	36	36	36	72	72	36	56	36	18	18	18

† : Pup was euthanased for general health reasons unrelated to vaccination

Grey shaded: Active seroconversion (>twofold rise in antibody titre). The first indication of an active immune response to vaccination is marked by the sudden disappearance of maternally derived antibody and a short timeframe of seronegativity that coincides with a vaccine virus viraemia and precedes a rapid active antibody response.

TABLE S1B. MDA Efficacy Study Serum Neutralisation (SN) Serological Data

Group	Pup ID	Days Post Vaccination																	
		0			4			7			11			14			18		
		2a	2b	2c	2a	2b	2c	2a	2b	2c	2a	2b	2c	2a	2b	2c	2a	2b	2c
1 Vaccinates	**8732**	3200	2016	2016	1008	4032	1131	635	1796	1008	640	1016	905	453	751	545	403	254	226
	**8995**	806	718	806	508	905	905	359	806	640	376	905	320	No Sample			36,204	36,204	51,200
	**9019**	905	403	905	453	905	806	453	806	640	453	806	508	≤14	≤14	≤14	18,102	21,816	21,816
	**8651**	905	718	905	545	1091	806	160	320	320	3805	1878	4755	2363	12,800	6400	No Sample		
	**8580**	905	905	1016	718	905	806	≤14	≤14	≤14	1903	9051	2263	†					
	**9030**	905	806	905	403	1280	905	453	905	806	453	640	254	18,102	36,204	18,102	≥129,016	≥129,016	102,400
	**8584**	1280	640	1280	718	1613	905	≤14	≤14	≤14	2263	6400	2263	5454	9051	8063	18,102	32,254	21,816
	**9002**	2540	3200	4032	1363	4032	1363	1131	2263	1600	905	1613	1280	508	1016	640	806	320	508
	**8998**	4032	2263	2263	1363	3200	1600	1131	2263	1270	320	202	403	5454	15,020	12,800	32,254	20,319	25,600
	**8635**	4032	1878	1878	1131	4525	2540	1270	2263	2263	800	2263	1131	504	2016	635	806	508	453
	**8755**	4525	3200	4525	2540	4032	3200	2016	5080	2016	1181	3200	1600	800	2263	1270	453	320	453
2 Controls	**9033**	545	226	640	453	905	751	273	508	403	202	751	226	160	453	376	101	32	80
	**9024**	806	538	905	508	1280	806	403	640	640	453	905	453	320	453	320	202	101	226
	**8747**	4032	2540	4032	1008	4525	2540	1008	3200	1600	640	1810	1280	473	1613	1613	453	160	453
	**8582**	4032	3200	4032	1131	3200	2016	1363	3200	2263	686	2540	1131	800	2016	1270	806	376	453
	**8751**	3200	2016	2727	1181	2263	1600	1131	3200	1008	1810	1613	1280	453	1810	1613	403	254	320

Group	Pup ID	Days Post Vaccination														
		21			25			28			31			34		
		2a	2b	2c	2a	2b	2c	2a	2b	2c	2a	2b	2c	2a	2b	2c
	8732	453	188	453	403	254	226	469	317	317	25,600	12,800	30,444	30,041	20,319	20,319
	8995	32,254	36,204	18,102	36,204	36,204	51,200	36,204	64,508	64,508	20,319	32,254	36,204	36,204	32,254	51,200
	9019	21,816	20,319	20,319	18,102	21,816	21,816	18,102	18,102	12,800	16,127	16,127	10,159	10,159	25,600	20,319
	8651	30,041	30,041	25,600		No Sample		32,254	32,254	36,204	36,204	32,254	32,254	18,102	36,204	43,632
	8580								†							
1 Vaccinates	9030	64,508	36,204	51,200	≥129,016	≥129,016	102,400	≥144,815	≥144,815	≥144,815	64,508	72,408	51,200	72,408	72,408	64,508
	8584	16,127	21,816	25,600	18,102	32,254	21,816	32,254	36,204	40,637	36,204	30,041	32,254	36,204	40,637	51,200
	9002	640	806	508	806	320	508	545	180	403	188	127	254	226	160	320
	8998	16,127	18,102	32,254	32,254	20,319	25,600	32,254	32,254	32,254	25,600	20,319	32,254	25,600	32,254	51,200
	8635	1016	640	905	806	508	453	453	202	453	254	254	273	226	160	226
	8755	905	640	806	453	320	453	453	160	453	320	226	202	254	188	202
	9033	160	68	202	101	32	80	63	28	80	50	40	57	40	57	50
	9024	226	118	254	202	101	226	127	68	160	101	57	113	80	101	101
2 Controls	8747	806	254	453	453	160	453	453	160	226	226	160	202	160	160	127
	8582	806	806	806	806	376	453	403	226	508	160	160	320	226	160	127
	8751	640	403	403	403	254	320	403	226	226	202	160	202	160	136	160

Group	Pup ID	Days Post Vaccination														
		38			41			45			48			52		
		**2a**	**2b**	**2c**	**2a**	**2b**	**2c**	**2a**	**2b**	**2c**	**2a**	**2b**	**2c**	**2a**	**2b**	**2c**
	**8732**	15,020	20,319	36,204	12,800	12,800	12,800	10,763	25,600	16,127	40,637	25,600	25,600	36,204	25,600	64,508
	**8995**	36,204	64,508	51,200	36,204	36,204	40,637	36,204	72,408	64,508	51,200	36,204	40,637	64,508	36,204	64,508
	**9019**	36,204	36,204	36,204	30,041	32,254	51,200	25,600	25,600	32,254	25,600	20,319	36,204	36,204	36,204	40,637
	**8651**	57,470	64,508	64,508	51,200	≥144,815	87,263	51,200	72,408	81,275	72,408	64,508	87,263	72,408	64,508	102,400
**1 Vaccinates**	**8580**								†							
	**9030**	64,508	36,204	64,508	40,637	≥144,815	72,408	51,200	72,408	40,637	72,408	30,041	51,200	40,637	30,444	64,508
	**8584**	32,254	64,508	51,200	36,204	≥144,815	40,637	36,204	57,470	72,408	36,204	40,637	64,508	40,637	57,470	64,508
	**9002**	160	113	160	101	127	113	101	80	101	50	≤14	40	10,908	10,159	10,159
	**8998**	32,254	32,254	36,204	15,020	25,600	32,254	18,102	32,254	32,254	32,254	36,204	40,637	25,600	20,319	32,254
	**8635**	226	113	202	≤14	≤14	≤14	16,127	25,600	32,254	25,600	12,800	25,600	36,204	14,368	28,735
	**8755**	226	160	160	202	202	226	113	68	113	63	≤15	57	9051	3200	14,368
	**9033**	40	40	45	28	50	28	25	25	25	25	≤14	≤14	≤14	≤14	≤16
	**9024**	80	80	101	57	101	57	50	50	50	40	≤18	50	28	28	32
**2 Controls**	**8747**	127	101	127	113	113	80	63	63	63	57	34	50	80	28	50
	**8582**	160	113	160	136	136	160	80	113	113	113	59	80	101	57	113
	**8751**	113	80	127	50	63	80	63	40	57	63	45	50	40	28	40

† : Pup was euthanased for general health reasons that were unrelated to vaccination

Grey shaded: Active seroconversion (>twofold rise in antibody titre). The first indication of an active immune response to vaccination is marked by the sudden disappearance of maternally derived antibody and a short timeframe of seronegativity that coincides with a vaccine virus viraemia and precedes a rapid active antibody respon
